# Systematic review of clinical evidence on postoperative delirium: literature search of original studies based on validated diagnostic scales

**DOI:** 10.1186/s44158-021-00021-8

**Published:** 2021-11-25

**Authors:** F. Bilotta, G. Russo, M. Verrengia, A. Sportelli, L. Foti, G. Villa, S. Romagnoli

**Affiliations:** 1 Department of Anesthesiology and Intensive Care Medicine, Policlinico Umbero I, “Sapienza”, University of Rome, Rome, Italy; 2 Department of Health Science section of Anesthesiology and Intensive Care, University of Florence, Florence, Italy; 3 Department of Anesthesiology and Intensive Care, Azienda Ospedaliero-Universitaria Careggi, Florence, Italy

**Keywords:** Postoperative delirium, Validated diagnostic postoperative delirium scales, Confused assessment method, Guidelines, Level of evidence, Evidence-based medicine

## Abstract

**Background:**

Postoperative delirium is a serious complication that can occur within the 5th postoperative day. In 2017, the European Society of Anesthesiologists delivered dedicated guidelines that reported the need for routine monitoring using validated scales.

**Objective:**

Aim of this systematic review is to identify clinical studies related to postoperative delirium that included postoperative monitoring with validated scales.

**Design:**

Systematic review

**Methods:**

Searched keywords included the following terms: postoperative, postsurgical, post anesthesia, anesthesia recovery, delirium, and confusion. Two researchers independently screened retrieved studies using a data extraction form.

**Results:**

Literature search led to retrieve 6475 hits; of these, 260 studies (5.6% of the retrieved), published between 1987 and 2021, included in their methods a diagnostic workup with the use of a postoperative delirium validated scale and monitored patients for more than 24 h, therefore are qualified to be included in the present systematic review.

**Conclusion:**

In conclusion, available clinical literature on postoperative delirium relies on a limited number of studies, that included a validated diagnostic workup based on validated scales, extracted from a large series of studies that used inconsistent diagnostic criteria. In order to extract indications based on reliable evidence-based criteria, these are the studies that should be selectively considered. The analysis of these studies can also serve to design future projects and to test clinical hypothesis with a more standardized methodological approach.

## Editor’s key points

- Clinical studies on postoperative delirium (POD) that rely on validated diagnostic scales are a fraction of the available literature accounting for only 5.6% of retrieved studies. This systematic review intended to provide the full list of studies that used a validated POD diagnostic scale.
- Studies that fulfill inclusion criteria for the present systematic review can provide clinical and research insights for evidence-based prevention, diagnosis, and treatment of POD.
- Of the 12 scales listed by the ESA-POD-GL, the 260 original studies included in this systematic review (SR), 205/260 (78.8%) adopted a single POD diagnostic scale, 48/260 (18.5%) used 2 diagnostic scales, and 7/260 (2.7%) used 3 or more diagnostic scales. The Confusion Assessment Method (CAM), CAM in Intensive Care Unit (CAM-ICU), and Diagnostic and Statistical Manual of mental disorders (DSM) accounted for more than 95% of used scales.

## Introduction

Postoperative delirium (POD) is an acute and fluctuating alteration of mental state of reduced awareness and disturbance of attention that can occur immediately after the recovery from anesthesia and up to 5 postoperative days [1]. It is reported to complicate the postoperative course of a substantial proportion of patients and is diagnosed in 10–50% patients depending on the specific clinical setting including age cohort, type of surgery (e.g., abdominal, orthopedic, urological, thoracic), and the indication criteria (elective, emergency, or urgent) [2, 3]. Clinical presentation of POD includes hyperactivity, hypoactivity, and mixed forms, and its occurrence is associated with increased perioperative morbidity and prolonged hospitalization, worse functional outcome and survival rates to long-term follow-up [4, 5].

In 2017, the task force of the European Society of Anesthesia and Intensive Care (ESAIC-TF) on POD delivered dedicated guidelines that reported the need to implement routine monitoring using validated scales (Table 1) [1]. These guidelines highlight the need to engage with “[ … ] integrated actions aimed to reduce the incidence and duration of POD.”; nevertheless, appropriate initiatives can only be identified using evidence-based medicine principles [6–8]. The prerequisite for data analysis is the selection of studies that fulfill necessary quality criteria to be considered for structured recommendations, including appropriate diagnostic workup.

**Table 1:** Scales validated for POD monitoring listed in the European Society of Anesthesia and Intensive Care (ESAIC) guidelines [1]

POD validated scale	
■ Diagnostic and Statistical Manual of Mental Disorders Fifth Edition (DSM-5); ■ 10th revision of the International Statistical Classification of Diseases and Related Health Problems (ICD 10); ■ Nursing Delirium Screening Scale (Nu-DESC); ■ Confusion Assessment Method (CAM); CAM-ICU; ■ Delirium Rating Scale-98; ■ Memorial Delirium Assessment Scale; ■ Bedside Confusion Scale; ■ Clinical Assessment of Confusion; ■ Confusion Rating Scale; ■ Delirium-O-Meter; ■ Delirium Observation Screening; ■ Delirium symptom interview (DSI); ■ Neelon and Champagne Confusion Scale (NEECHAM); ■ 4 ‘A’s Test	

The aim of this systematic review (SR) is to identify clinical studies related to POD that included a postoperative monitoring diagnostic workup based on validated scales.

## Methods

This SR was registered in the International Prospective Register of Systematic Review (PROSPERO registration number: CRD42021246906) and performed in accordance with the Preferred Reporting Items for Systematic Reviews and Meta-analyses (PRISMA) recommendations [9]. Literature search strategy was based on the 2017 ESAIC-TF POD guidelines; the same keywords were used and to optimize data retrieving; query strings were updated conforming the most recent version of searched databases (PubMed, Embase, Cinhal, Cochrane, Scopus, and Web of science) [1]. Searched keywords included the following terms: postoperative, postsurgical, post anesthesia, anesthesia recovery, delirium, and confusion. Literature search strings, tailored to individual requirements of each of the searched database, were used and are listed in Appendix 1. Considering the enhancement of “automatic term mapping”, implemented in PubMed 2019–delivered version, the use of the asterisk was avoided. Redundancies of the strings used by the ESAIC-TF on POD for literature search accomplished for guidelines released in 2017 have been eliminated, and inconsistencies have been corrected. Furthermore, references sections of retrieved SRs and meta-analyses were searched for studies missed through database literature screening. Literature exploration was accomplished between April and May 2021 and intended to include evidence published up to March 15th, 2021. After automatic and manual duplicate removal, selected papers were evaluated throughout two screening phases.

### Study selection and inclusion criteria

Suitable studies included: randomized clinical trials (RCTs), prospective and retrospective studies, cohort studies, and case–control studies. The adult population (older than 18 years old) was tested or monitored in the postoperative period for more than 24 h with a validated POD scale. Studies designed to record POD as primary or secondary outcome, in patients undergoing non-cardiac and non-cerebral surgery have been selectively included. Only full papers in English language were considered eligible. Papers that did not report information on an original dataset of patients (editorials, commentaries, reviews, metanalysis, etc.) and case series reporting < 5 cases were excluded. Abstracts and meeting/symposium proceedings were excluded. Studies that selectively recruited patients presenting POD were not considered appropriate for this SR. Online registered protocols of trials (ClinicalTrials.gov, WHO) were excluded.

### First screening phase

Two researchers (GR and LF) independently screened and assessed title, details of the publication, and the abstract, to identify papers that fulfilled the following criteria: full paper in English, that present original information on adult patients who have undergone surgery (except cardiac or brain surgery), and in which POD recording was among the primary or secondary outcomes.

### Second screening phase

Studies that qualified for the second screening phase were evaluated throughout full-text analysis of type and timing of POD monitoring. Only those that used a POD validated scale (Table 1), for more than 24 h, in patients not selectively presenting delirium, and that reported the quantitative figure of recorded POD incidence were considered eligible. Selected studies were analyzed using a standardized data extraction form, and the following variables were extrapolated: validated diagnostic POD scale used, number of recruited patients, overall POD incidence, study design and structure (i.e., RCTs, prospective or retrospective observational, and single center or multicentric), type of surgery (categorized as: breast, eye, general abdominal, gynecologic, maxillofacial, orthopedic—not spine, otolaryngologic, plastic, spinal, thoracic, urologic, vascular), and country where the study was conducted. Data will be reported and made available in the SRDR website.

## Results

### Study identification

**Fig. 1:**
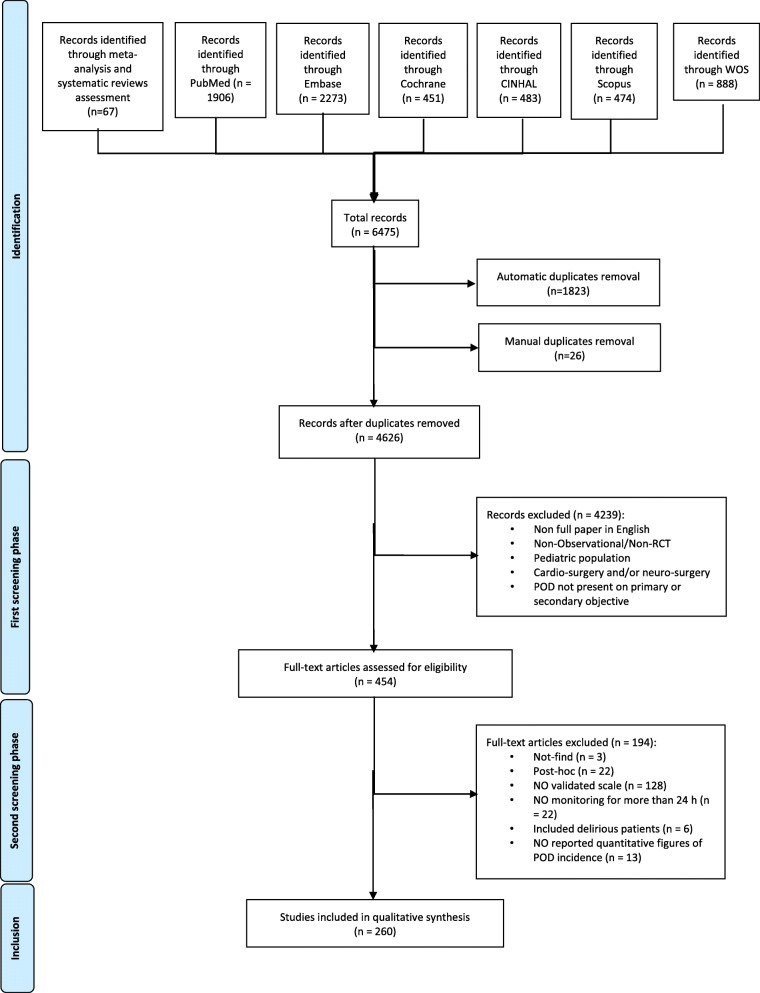
CONSORT diagram

Database search led to retrieve 6475 hits; after duplicate removal (1849), 4626 qualified for subsequent evaluation and underwent the first-phase screening (Fig. 1). A total of 4239 studies were excluded during the first screening phase (not full-text or original studies or not published in English or that included patients aged < 18 years or who had undergone brain or cardiac procedures). The analysis of references sections of retrieved SRs and meta-analyses led to identify additional 67 studies not retrieved through database literature search but are suitable for second-phase screening. This process led to select 454 original studies that fulfilled the criteria for second-phase screening. Among studies screened in the second phase, it was not possible to retrieve the full text of 3 manuscripts; 22 were post hoc analyses of trials included in the list. Of the 429 studies analyzed, 128 did not use a POD validated scale; in the 22 that used POD monitoring that lasted < 24 h, 6 selectively included patients presenting delirium, and 13 did not report POD incidence quantitatively. A total of 260 studies (5.6% of the studies retrieved through literature search), published between 1987 and 2021, included in their methods a diagnostic workup with the use of a POD validated scale and monitored patients for more than 24 h therefore are qualified to be included in the present SR; the full list of these papers is in Appendix 2.

### Used diagnostic scales

The 260 original studies included in this SR utilized some of the 12 scales listed by the ESA-POD-GL (Table 1): 205/260 (78.8%) adopted a single POD diagnostic scale, 48/260 (18.5%) used 2 diagnostic scales, and 7/260 (2.7%) used 3 or more diagnostic scales. The Confusion Assessment Method (CAM; versions: 3D, b, S, CR, 4) was the most frequently used diagnostic scale and was included in 158/260 (60.8%), the CAM-Intensive Care Unit (ICU) in 38/260 (14.6%), the Diagnostic and Statistical Manual of Mental Disorders (DSM) (versions: III, IV, V) in 58/260 (22.3%), the International Classification of Disease (ICD) (versions 9, 10) in 5/260 (2.0%), the Delirium Rating Scale (DSR) (R-98 or the previous) in 20/260 (7.7%), the Nursing Delirium Screening Scale (Nu-DESC) in 11/260 (4.2 %), the Memorial Delirium Assessment Scale (MDAS) in 9/260 (3.4%), the Delirium observation screening in 13/260 (5.0%), the Neelon and Champagne Confusion Scale (NEECHAM) in 5/260 (2.0%), the Delirium Symptom Interview (DSI) in 3/260 (1.1%), and the 4 'A's (Arousal, Attention, Abbreviated Mental Test - 4, Acute change) Test in 1/260 (0.4%).

### Number of recruited patients

Data from a total of 1,327,808 patients were reported in the 260 studies included in this SR. The number of recruited patients ranged between 11 and 578,457; the mode (value that appears more often) is 101 patients. Considering the quartile distribution, 25% of studies include 11–89 patients (mean value: 56.3); 25%, 90–184 (mean value: 124.7); 25%, 186–366 (mean value: 256.1); and 25%, 377–578.457 (mean value: 19.986.7).

### Overall POD incidence

Among the 260 studies that qualified for this SR, the reported POD incidence ranged between 0.01 and 84.0%; mean POD incidence was 23.0%, and the mode was 25.0%. Considering the quartile distribution, in 25% of the studies, POD overall incidence ranged from 0.01 to 13.1% (mean value: 8.0%); 25%, 13.5–21.1% (mean value: 17.1%); 25%, 21.2–27.9% (mean value: 24.3%); and 25%, 28.0–84.0% (mean value: 41.3%). In patients aged > 65, reported POD incidence ranged between 15 and 40% (15%, 32%, 40%) [10–12].

### Study design and structure

Of the 260 studies selected in this SR, 156 were single-center, and 104 were multicentric; of these, 118 were prospective observational, 109 RCTs, and 33 retrospective observational studies.

### Type of surgery

The 260 studies included in this SR reported data from patients undergoing a single and selective type of surgery in 223/260 (85.8%), while 35/260 (13.5%) reported data from two or multiple types of surgical procedures; 2/260 (0.8%) included patients undergoing non-brain and non-cardiac surgery, without specifying the exact type of surgery.

Considering the exclusion of cardiac and brain surgery in the first phase of screening, the most common surgery was orthopedic with 126/260 (48.4%); general abdominal surgery with 109/260 (42.0%); thoracic with 23/260 (8.8%); urological with 24/260 (9.2%); spinal with 20/260 (7.7%); maxillofacial with 7/260 (2.7%); breast with 1/260 (0.4%); otolaryngologic with 6/260 (2.3%); vascular with 8/260 (3.0%); gynecological with 9/260 (3.5%); plastic with 1/260 (0.4%), and eye with 1/260 (0.4%).

### Country where the studies were conducted

The country with the highest number of original studies was China: 59/260 (22.3%); the USA was the second one: 58/260 (22.3%). Among the multicenter studies, 6/104 were international cooperative studies. One study was included from each of the following countries: Albania, Brazil, Chile, Finland, Israel, Malesia, New Zealand, and Serbia; 2 studies each from Iran, Switzerland, and Turkey; 3 studies each from Denmark, Greece, Portugal, and Thailand; 4 studies each from Australia, Belgium, Spain, and Taiwan; 5 studies from France; 6 studies each from Canada and Norway; 7 studies from Sweden; 9 studies from Italy; 10 studies from the UK, 17 studies each from Korea and Germany; 18 studies from Netherlands; and 22 studies from Japan. Even when report data were taken from similar surgical settings, studies completed in different countries display no homogeneous figures (orthopedic surgery: the USA, the UK,Turkey; Urologic surgery: China, Italy, Germany) [13–18].

### POD risk factors

The analysis of the studies that qualified for the present SR suggests that there are risk factors that specifically connect with POD. Some of these risk factors can be ruled out in the preoperative phase (age; cognitive status; level of education; type and indication criteria for surgery; preoperative pain severity; some laboratory test exams; individual habits: chronic benzodiazepine use, alcohol and drug abuse, and low physical activity; etc.); other factors can complicate the intra or postoperative course (severe bleeding, using opioid, anemization, transfusion, ICU admission, hypothermia (core temperature < 36 °C at admission to the recovery room); etc.).

## Discussion

The primary endpoint of this SR is to provide a selection of clinical studies that addressed POD including in their methods a postoperative monitoring diagnostic workup based on validated scales. The full list of these studies—reported as an appendix to this manuscript—can serve clinicians and researchers to extract methodological hints and available evidence-based clues. The analysis of used POD diagnostic scales indicates that the largest majority of the studies was used a single scale and that CAM and CAM-ICU are the most used. The analysis of the studies that fulfilled the criteria to be listed in the present SR demonstrated the high heterogeneity of the number of recruited patients. Of note, considering the absolute value, the recorded results should adequately support the identification of pre, intra, and postoperative risk factors; stratification criteria; and preventive and therapeutic protocols. The reported POD incidence ranged widely but consistently indicated that, in patients aged > 65 years, this complication is frequent and occurs in about a quarter of the cases thus making it a priority in perioperative medicine. The most represented type of study is prospective observational tightly followed by RCTs witnessing the interest and the effort that the scientific community is investing to better understand the pathophysiologic mechanisms and therapeutic strategies that can effectively be implemented to prevent and treat POD. Several therapies had been tested, but available evidence is conflicting and inconclusive. Presented cases were selectively recruited among single types of surgery, most commonly orthopedic or general abdominal. Interestingly, studies accomplished in China or the USA are equally represented and account for over 50% of the total.

The need to standardize diagnostic criteria is an emerging necessity in studies intended to evaluate postoperative neurocognitive complications. This is also proved by the recent systematic review on POCD published by Borchers *et al.* that highlights how the heterogeneity in the diagnostic workup can bias extracted evidence [19]. The authors compared the methodology of studies on postoperative cognitive decline to the reference criteria published in 1995 and, similarly to our findings, from more than 8000 studies published, only 274 (3.4%) used baseline cognitive testing and followed patients for a proper length of time. Noteworthy, according to a recent dedicated SR, the level of evidence that supports the largest proportion of guidelines released for anesthesiologists on perioperative care by the North American and European societies relies on a low level of evidence [20]. Hence, it is necessary to identify POD studies that fulfill quality criteria that can extract clinical and research insights.

In the present SR, clinical trials that involved cardiac or brain surgery patients were excluded because of the specific risk of cerebral dysfunction that can take place in these surgical settings. Patients undergoing cardiac surgery, with or without extracorporeal circulation, as well as those undergoing brain surgery, can develop focal ischemia and stroke that mask or trigger functional cognitive abnormalities [21–23].

The present literature analysis, despite using the same literature search strategy (including keywords and searched databases) proposed and validated by the ESA task force on POD, extracted a more limited number of hits. This is possibly due to the revised functional algorithm used by scientific library databases. Especially relevant is the finding that a significant number of clinical studies related to POD were not identified with the used keywords and strings. The mismatch between searching criteria and published evidence is possibly attributable to a suboptimal manuscript categorization during the submission and publication phases. In the future, it would be important to identify a clear nomenclature for those studies that provide insights related to POD. The present SR was designed conforming to the latest version of the “automatic term mapping” enhancement released by PubMed in 2019 and the use of the asterisk was removed by the literature search strings accordingly. Furthermore, redundancies of the strings used by the ESAIC-TF for literature search, accomplished to prepare the guidelines released in 2017, have been eliminated.

### Study limitations

The major limitation of this SR is that it does not provide any specific information on POD prevention or diagnosis, or treatment. This is because we intend the underlying work to provide to the scientific community a shared platform to extract evidence-based medicine principles and methodological hints to be included in future studies. Considering the large variability of anesthesiology techniques used and the non-homogeneity in reporting the adopted approach (general, neuraxial, or loco-regional) and of the various types of surgical procedures, it was not possible to describe, in the present SR, in detail the relationship between the type of used anesthesia and POD. Future analysis of the presented list can address several aspects including the sensibility and sensitivity of the POD diagnostic scales considering those studies that adopted two or more of the validated scales.

In conclusion, available clinical literature on POD, that qualifies to prepare evidence-based indications, relies on a limited selection of studies that include a diagnostic workup based on validated scales. In order to extract indications based on reliable evidence-based criteria, these are the studies that should be selectively considered. The analysis of these studies can also serve to design future projects and to test clinical hypothesis with a more standardized methodological approach.

## Appendix 1. Full list of literature search strings

1. PubMed:

(Delirium OR delirious OR confusion OR disorientation OR bewilderment) AND (Postoperative OR "postoperative" OR post intervention OR "post intervention" OR "post-surgical" OR postsurgical OR "post-surgery" OR post surgery OR "anesthesia recovery" OR "anesthesia recovery" OR "Anesthesia Recovery Period"[Mesh] OR post anesthesia OR "post anesthesia" OR "post anesthesia")

2. EMBASE:

((('delirium'/exp OR delirium OR delirious OR 'confusion'/exp OR confusion OR 'disorientation'/exp OR disorientation OR bewilderment) AND ('postoperative complication'/exp OR postoperative OR 'post-operative' OR 'postoperative period'/exp OR post-intervention OR 'post intervention' OR 'post-surgical' OR postsurgical OR 'post-surgery' OR post-surgery OR 'anesthesia recovery'/exp OR 'anesthesia recovery' OR 'anesthesia recovery'/exp OR 'anesthesia recovery' OR post anesthesia OR 'post anesthesia' OR 'post anesthesia')) OR 'postoperative delirium'/exp) AND ([cochrane review]/lim OR [systematic review]/lim OR [meta-analysis]/lim OR [randomized controlled trial]/lim OR 'observational study' OR 'case study')

3. CINAHL:

(Delirium OR delirious OR confusion OR disorientation OR bewilderment) AND (Postoperative OR "post-operative" OR post intervention OR "post intervention" OR "post-surgical" OR postsurgical OR "post-surgery" OR post-surgery OR "anesthesia recovery" OR "anesthesia recovery" OR post anesthesia OR "post anesthesia" OR "post anesthesia")

Limiters—Publication Type: Case Study, Meta-Analysis, Randomized Controlled Trial, Systematic Review

OR

((Delirium OR delirious OR confusion OR disorientation OR bewilderment) AND (Postoperative OR "post-operative" OR post intervention OR "post intervention" OR "post-surgical" OR postsurgical OR "post-surgery" OR post-surgery OR "anesthesia recovery" OR "anesthesia recovery" OR post anesthesia OR "post anesthesia" OR "post anesthesia")) AND (observational study OR observational research)

OR

(MH "Delirium" OR MH "Confusion+") AND ((MH "Postoperative Complications") OR (MH "Postoperative Period"))

Limiters—Publication Type: Case Study, Meta-Analysis, Randomized Controlled Trial, Systematic Review

OR

(MH "Delirium" OR MH "Confusion+") AND ((MH "Postoperative Complications") OR (MH "Postoperative Period")) AND ((observational study OR observational research))

4. COCHRANE:

#1 ((Delirium OR delirious OR confusion OR disorientation OR bewilderment) AND (Postoperative OR "post-operative" OR post intervention OR "post intervention" OR "post-surgical" OR postsurgical OR "post-surgery" OR post-surgery OR "anesthesia recovery" OR "anesthesia recovery" OR post anesthesia OR "post anesthesia" OR "post anesthesia")):ti,ab,kw 1829

#2 MeSH descriptor: [Delirium] explode all trees 765

#3 MeSH descriptor: [Confusion] explode all trees 899

#4 #2 OR #3 899

#5 MeSH descriptor: [Postoperative Period] explode all trees 5872

#6 MeSH descriptor: [Postoperative Complications] explode all trees 39635

#7 MeSH descriptor: [Anesthesia Recovery Period] explode all trees 2031

#8 #5 OR #6 OR #7 43861

#9 #4 AND #8 326

#10 #1 OR #9 1853

5. SCOPUS:

TITLE-ABS (delirium OR delirious OR confusion OR disorientation OR bewilderment) AND TITLE-ABS (postoperative OR {post-operative} OR post intervention OR {post intervention} OR {post-surgical} OR postsurgical OR {post-surgery} OR post-surgery OR {anesthesia recovery} OR {anesthesia recovery} OR post anesthesia OR {post anesthesia} OR {post anesthesia}) AND TITLE-ABS ({systematic review} OR {case series} OR {randomized controlled trial} OR rct OR {meta-analysis} OR metanalysis OR {observational study})

6. WEB OF SCIENCE:

TITLE-ABS (delirium OR delirious OR confusion OR disorientation OR bewilderment) AND TITLE-ABS (postoperative OR {post-operative} OR post intervention OR {post intervention} OR {post-surgical} OR postsurgical OR {post-surgery} OR post-surgery OR {anesthesia recovery} OR {anesthesia recovery} OR post anesthesia OR {post anesthesia} OR {post anesthesia}) AND TITLE-ABS ({systematic review} OR {case series} OR {randomized controlled trial} OR rct OR {meta-analysis} OR metanalysis OR {observational study})

## Appendix 2. List of the studies that qualified for systematic review

1. Berggren D, Gustafson Y, Eriksson B, et al. Postoperative confusion after anesthesia in elderly patients with femoral neck fractures. *Anesth Analg*; 1987; **66**: 497–504

2. Rogers MP, Liang MH, Daltroy LH, et al. Delirium after elective orthopedic surgery: risk factors and natural history. *Int J Psychiatry Med*; 1989; **19**: 109–21

3. Williams-Russo P, Urquhart BL, Sharrock NE, et al. Postoperative delirium - predictors and prognosis in elderly orthopedic patients. *J Am Geriatr Soc*; 1992; **40**: 759–67

4. DiMartini AF, Trzepacz PT, Pajer KA, Faett D, Fung J. Neuropsychiatric side effects of FK506 vs. cyclosporine A. First-week postoperative findings. *Psychosomatics*; 1997; **38**: 565–9

5. Lynch EPP, Lazor MAA, Gellis JEE, Orav J, Goldman L, Marcantonio ERR. The of impact postoperative pain on the development of postoperative delirium. *Anesth Analg*; 1998; **86**: 781–5

6. Kaneko T, Cai J, Ishikura T, Kobayashi M, Naka T, Kaibara N. Prophylactic consecutive administration of haloperidol can reduce the occurrence of postoperative delirium in gastrointestinal surgery. *Yonago Acta Med*; 1999; **42**: 179–84

7. Mann C, Pouzeratte Y, Boccara G, et al. Comparison of intravenous or epidural patient-controlled analgesia in the elderly after major abdominal surgery. *Anesthesiology*; 2000; **92**: 433–41

8. Milisen K, Foreman MD, Abraham IL, et al. A nurse-led interdisciplinary intervention program for delirium in elderly hip-fracture patients. *J Am Geriatr Soc*; 2001; **49**: 523-532

9. Marcantonio ER, Flacker JM, Wright RJ, Resnick NM. Reducing delirium after hip fracture: a randomized trial. *J Am Geriatr Soc*; 2001; **49**: 516–22

10. Aizawa K, Kanai T, Saikawa Y, et al. A novel approach to the prevention of postoperative delirium in the elderly after gastrointestinal surgery. *Surg Today*; 2002; **32**: 310–4

11. Kudoh A, Katagai H, Takazawa T. Anesthesia with ketamine, propofol, and fentanyl decreases the frequency of postoperative psychosis emergence and confusion in schizophrenic patients. *J Clin Anesth*; 2002; **14**: 107–10

12. Kudoh A, Katagai H, Takazawa T. Antidepressant treatment for chronic depressed patients should not be discontinued prior to anesthesia. *Can J Anesth*; 2002; **49**: 132–6

13. Kudoh A, Takase H, Takahira Y, Katagai H, Takazawa T. Postoperative confusion in schizophrenic patients is affected by interleukin-6. *J Clin Anesth*; 2003; **15**: 455–62

14. Kudoh A, Takase H, Takazawa T. A comparison of anesthetic quality in propofol-spinal anesthesia and propofol-fentanyl anesthesia for total knee arthroplasty in elderly patients. *J Clin Anesth*; 2004; **16**: 405–10

15. Kudoh A, Katagai H, Takase H, Takazawa T. Effect of preoperative discontinuation of antipsychotics in schizophrenic patients on outcome during and after anaesthesia. Eur. J. Anaesthesiol; 2004. p. 414–6

16. Nishikawa K, Nakayama M, Omote K, Namiki A. Recovery characteristics and post-operative delirium after long-duration laparoscope-assisted surgery in elderly patients: propofol-based vs. sevoflurane-based anesthesia. *Acta Anaesthesiol Scand*; 2004; **48**: 162–8

17. Laki A, Garb JL, Fingeroth R, Krushell R. Donepezil in the prevention and treatment of post-surgical delirium. *Am J Geriatr Psychiatry*; 2005; **13**: 1100–6

18. Kalisvaart KJ, de Jonghe JFM, Bogaards MJ, et al. Haloperidol prophylaxis for elderly hip-surgery patients at risk for delirium: a randomized placebo-controlled study. *J Am Geriatr Soc*; 2005; **53**: 1658–66

19. Pitkala K, Laurila J, Strandberg TE, Tilvis R. Prevention and treatment of postoperative. *Psychogeriatrics;* 2005; **17**: 147

20. Papaioannou A, Fraidakis O, Michaloudis D, Balalis C, Askitopoulou H. The impact of the type of anaesthesia on cognitive status and delirium during the first postoperative days in elderly patients. *Eur J Anaesthesiol*; 2005; **22**: 492–9

21. Contín AM, Perez-Jara J, Alonso-Contín A, Enguix A, Ramos F. Postoperative delirium after elective orthopedic surgery. *Int J Geriatr Psychiatry*; 2005; **20**: 595–7

22. Freter SH, Dunbar MJ, MacLeod H, Morrison M, MacKnight C, Rockwood K. Predicting post-operative delirium in elective orthopaedic patients: the Delirium Elderly At-Risk (DEAR) instrument. Age Ageing; 2005; p. 169–71

23. Stenvall M, Olofsson B, Lundstrom M, Svensson O, Nyberg L, Gustafson Y. Inpatient falls and injuries in older patients treated for femoral neck fracture. *Arch Gerontol Geriatr*; 2006; **43**: 389-399

24. Leung JM, Sands LP, Rico M, et al. Pilot clinical trial of gabapentin to decrease postoperative delirium in older patients. *Neurology*; 2006; **67**: 1251–3

25. Beaussier M, Weickmans H, Parc Y, et al. Postoperative analgesia and recovery course after major colorectal surgery in elderly patients: a randomized comparison between intrathecal morphine and intravenous PCA morphine. *Reg Anesth Pain Med*; 2006; **31**: 531–8

26. McCaffrey R, Locsin R. The effect of music on pain and acute confusion in older adults undergoing hip and knee surgery. *Holist Nurs Pract*; 2006; **20**: 216–8

27. Nishikawa K, Kimura S, Shimodate Y, Igarashi M, Namiki A. A comparison of intravenous-based and epidural-based techniques for anesthesia and postoperative analgesia in elderly patients undergoing laparoscopic cholecystectomy. *J Anesth*; 2007; **21**: 1–6

28. Sampson EL, Raven PR, Ndhlovu PN, et al. A randomized, double-blind, placebo-controlled trial of donepezil hydrochloride (Aricept) for reducing the incidence of postoperative delirium after elective total hip replacement. *Int J Geriatr Psychiatry*; 2007; **22**: 343–9

29. Ganai S, Lee KFF, Merrill A, et al. Adverse outcomes of geriatric patients undergoing abdominal surgery who are at high risk for delirium. *Arch Surg*; 2007; **142**: 1072–8

30. Taguchi T, Yano M, Kido Y. Influence of bright light therapy on postoperative patients: a pilot study. *Intensive Crit care Nurs*; 2007; **23**: 289–97

31. Lundstrom M, Olofsson B, Stenvall M, et al. Postoperative delirium in old patients with femoral neck fracture: a randomized intervention study. *Aging Clin Exp Res*; 2007; **19**: 178–86

32. Schrader SLP, Wellik KE, Demaerschalk BM, Caselli RJ, Woodruff BK, Wingerchuk DM. Adjunctive haloperidol prophylaxis reduces postoperative delirium severity and duration in at-risk elderly patients. *Neurologist*; 2008; **14**: 134–7

33. Da Cunha PTS, Artifon AN, Lima DP, et al. Hip fractures in the elderly: surgical treatment timing and its correlation with delirium and infection | Fratura de quadril em idosos: Tempo de abordagem cirúrgica e sua associação quanto a delirium e infecção. *Acta Ortop Bras;* 2008; **16**: 173–6

34. Robinson TNN, Raeburn CDD, Angles EMM, Moss M. Low tryptophan levels are associated with postoperative delirium in the elderly. *Am J Surg*; 2008; **196**: 670–4

35. Gao R, Yang ZZ, Li M, Shi ZC, Fu Q. Probable risk factors for postoperative delirium in patients undergoing spinal surgery. *Eur Spine J*; 2008; **17**: 1531–7

36. Koebrugge B, Koek HL, Van Wensen RJA, Dautzenberg PLJ, Bosscha K. Delirium after abdominal surgery at a surgical ward with a high standard of delirium care: incidence, risk factors and outcomes. *Dig Surg*; 2009; **26**: 63–8

37. Larsen KA, Kelly SE, Stern TA, et al. Administration of olanzapine to prevent postoperative delirium in elderly joint-replacement patients: a randomized, controlled trial. *Psychosomatics*; 2010; **51**: 409–18

38. Radtke FM, Franck M, MacGuill M, et al. Duration of fluid fasting and choice of analgesic are modifiable factors for early postoperative delirium. *Eur J Anaesthesiol*; 2010; **27**: 411–6

39. Radtke FM, Hagemann L, Franck M, Griesshaber M, Spies CD. Inadequate emergence and early postoperative delirium after anesthesia. *Anesth Analg*; 2010; **110**: S183

40. Ranhoff AH, Holvik K, Martinsen MI, Domaas K, Solheim LF. Older hip fracture patients: three groups with different needs. *BMC Geriatr*; 2010; **10**: 65

41. Tei M, Ikeda M, Haraguchi N, et al. Risk factors for postoperative delirium in elderly patients with colorectal cancer. *Surg Endosc*; 2010; **24**: 2135–9

42. Brouquet A, Cudennec T, Benoist S, et al. Impaired mobility, ASA status and administration of tramadol are risk factors for postoperative delirium in patients aged 75 years or more after major abdominal surgery. *Ann Surg*; 2010; **251**: 759–65

43. van Munster BC, Bisschop PH, Zwinderman AH, et al. Cortisol, interleukins and S100B in delirium in the elderly. *Brain Cogn*; 2010; **74**: 18–23

44. Lee JK, Park YS. Delirium after spinal surgery in Korean population. *Spine (Phila Pa 1976)*; 2010; **35**: 1729–32

45. Jain FA, Brooks JO 3rd, Larsen KA, et al. Individual risk profiles for postoperative delirium after joint replacement surgery. *Psychosomatics*; 2011; **52**: 410–6

46. Marcantonio ER, Palihnich K, Appleton P, Davis RB. Pilot randomized trial of donepezil hydrochloride for delirium after hip fracture. *J Am Geriatr Soc;* 2011; **59 Suppl 2**: S282-8

47. Tognoni P, Simonato A, Robutti N, et al. Re: Preoperative risk factors for postoperative delirium (POD) after urological surgery in the elderly. *J Urol*; 2011; **186**: 2302–3

48. Ono H, Taguchi T, Kido Y, Fujino Y, Doki Y. The usefulness of bright light therapy for patients after oesophagectomy. *Intensive Crit care Nurs* Netherlands; 2011; **27**: 158–66

49. Chen CCH, Lin MT, Tien YW, Yen CJ, Huang GH, Inouye SK. Modified hospital elder life program: effects on abdominal surgery patients. *J Am Coll Surg*; 2011; **213**: 245–52

50. Patti R, Saitta M, Cusumano G, Termine G, Di Vita G. Risk factors for postoperative delirium after colorectal surgery for carcinoma. *Eur J Oncol Nurs*; 2011; **15**: 519–23

51. Mangnall LT, Gallagher R, Stein-Parbury J. Postoperative delirium after colorectal surgery in older patients. *Am J Crit Care*; 2011; **20**: 45–55

52. Lee HJ, Hwang DS, Wang SK, Chee IS, Baeg S, Kim JL. Early assessment of delirium in elderly patients after hip surgery. *Psychiatry Investig*; 2011; **8**: 340–7

53. Jankowski CJ, Trenerry MR, Cook DJ, et al. Cognitive and functional predictors and sequelae of postoperative delirium in elderly patients undergoing elective joint arthroplasty. *Anesth Analg*; 2011; **112**: 1186–93

54. Wang W, Li H-LH-LH-L, Wang D-XD-XD-X, et al. Haloperidol prophylaxis decreases delirium incidence in elderly patients after noncardiac surgery: a randomized controlled trial*. *Crit Care Med*; 2012; **40**: 731–9

55. Do T-D, Lemogne C, Journois D, Safran D, Consoli SMM. Low social support is associated with an increased risk of postoperative delirium. *J Clin Anesth*; 2012; **24**: 126–32

56. Deschodt M, Braes T, Flamaing J, et al. Preventing delirium in older adults with recent hip fracture through multidisciplinary geriatric consultation. *J Am Geriatr Soc*; 2012; **60**: 733–9

57. Cerejeira JMSMS, Nogueira V, Luís P, Vaz-Serra A, Mukaetova-Ladinska EBB. The cholinergic system and inflammation: common pathways in delirium pathophysiology. *J Am Geriatr Soc*; 2012; **60**: 669–75

58. Papathanakos G, Pouagare M, Tzimas P, Arnaoutoglou E, Papadopoulos G. The impact of ondansetron on postoperative cognitive function in elderly patients undergoing surgery under general anaesthesia due to femoral fracture-preliminary data. *Eur J Anaesthesiol*; 2012; **29**: 29

59. Flink BJ, Rivelli SK, Cox EA, et al. Obstructive sleep apnea and incidence of postoperative delirium after elective knee replacement in the nondemented elderly. *Anesthesiology*; 2012; **116**: 788–96

60. Gani H, Domi R, Kodra N, et al. The incidence of postoperative delirium in elderly patients after urologic surgery. *Med Arh*; 2013; **67**: 45–7

61. Mézière A, Paillaud E, Belmin J, et al. Delirium in older people after proximal femoral fracture repair: role of a preoperative screening cognitive test. *Ann Fr Anesth Reanim*; 2013; **32**: e91-6

62. Radtke FM, Franck M, Lendner J, Krüger S, Wernecke KD, Spies CD. Monitoring depth of anaesthesia in a randomized trial decreases the rate of postoperative delirium but not postoperative cognitive dysfunction. *Br J Anaesth*; 2013; **110 Suppl**: i98-105

63. Hempenius L, Slaets JPJ, van Asselt D, de Bock GH, Wiggers T, van Leeuwen BL. Outcomes of a geriatric liaison intervention to prevent the development of postoperative delirium in frail elderly cancer patients: report on a multicentre, randomized, controlled trial. *PLoS One;* 2013; **8**: e64834

64. Yoshitaka S, Egi M, Morimatsu H, Kanazawa T, Toda Y, Morita K. Perioperative plasma melatonin concentration in postoperative critically ill patients: its association with delirium. *J Crit Care*; 2013; **28**: 236–42

65. Lescot T, Karvellas CJJ, Chaudhury P, et al. Postoperative delirium in the intensive care unit predicts worse outcomes in liver transplant recipients. *Can J Gastroenterol*; 2013; **27**: 207–12

66. Witlox J, Slor CJ, Jansen RWMM, et al. The neuropsychological sequelae of delirium in elderly patients with hip fracture three months after hospital discharge. *Int Psychogeriatrics*; 2013; **25**: 1521–31

67. Cerejeira J, Batista P, Nogueira V, Vaz-Serra A, Mukaetova-Ladinska EB. The stress response to surgery and postoperative delirium: evidence of hypothalamic-pituitary-adrenal axis hyperresponsiveness and decreased suppression of the GH/IGF-1 axis. *J Geriatr Psychiatry Neurol*; 2013; **26**: 185–94

68. Liu P, Li Y, Wang X, Zou X, Zhang D, Wang D, Li S. High serum interleukin-6 level is associated with increased risk of delirium in elderly patients after noncardiac surgery: a prospective cohort study. Chin Med J; 2013; 126(19): 3621-7.

69. Westhoff D, Witlox J, Koenderman L, et al. Preoperative cerebrospinal fluid cytokine levels and the risk of postoperative delirium in elderly hip fracture patients. *J Neuroinflammation*; 2013; **10**

70. Fineberg SJ, Nandyala S V., Marquez-Lara A, Oglesby M, Patel AA, Singh K. Incidence and risk factors for postoperative delirium after lumbar spine surgery. *Spine (Phila Pa 1976)*; 2013; **38**: 1790–6

71. Large MC, Reichard C, Williams JTB, et al. Incidence, risk factors, and complications of postoperative delirium in elderly patients undergoing radical cystectomy. *Urology*; 2013; **81**: 123–9

72. Zhang CH, Ma WQ, Yang YL, Dong FT, Wang HM, Wei HM. Effect of the intraoperative wake-up test in sevoflurane-sufentanil combined anesthesia during adolescent idiopathic scoliosis surgery: a randomized study. *J Clin Anesth*; 2013; **25**: 263–7

73. Jia Y, Jin G, Guo S, et al. Fast-track surgery decreases the incidence of postoperative delirium and other complications in elderly patients with colorectal carcinoma. *Langenbeck’s Arch Surg;* 2014; **399**: 77–84

74. Saczynski JSS, Inouye SKK, Kosar CMM, et al. Cognitive and brain reserve and the risk of postoperative delirium in older patients: analysis of data from a prospective observational study. *The Lancet Psychiatry*; 2014; **1**: 437–43

75. Chan MT V, Gin T. Delirium and cognitive decline after surgery: a randomised controlled trial of anaesthetic management to improve postoperative mental health outcome. *Hong Kong Med J*; 2014; **20 Suppl 7**: 28–9

76. Bellelli G, Mazzola P, Morandi A, et al. Duration of postoperative delirium is an independent predictor of 6-month mortality in older adults after hip fracture. *J Am Geriatr Soc*; 2014; **62**: 1335–40

77. Fukata S, Kawabata Y, Fujisiro K, et al. Haloperidol prophylaxis does not prevent postoperative delirium in elderly patients: a randomized, open-label prospective trial. *Surg Today*; 2014; **44**: 2305–13

78. Watne LO, Torbergsen AC, Conroy S, et al. The effect of a pre- and postoperative orthogeriatric service on cognitive function in patients with hip fracture: randomized controlled trial (Oslo Orthogeriatric Trial). *BMC Med;* 2014; **12**: 63

79. Robinson TN, Dunn CL, Adams JC, et al. Tryptophan supplementation and postoperative delirium--a randomized controlled trial. *J Am Geriatr Soc;* 2014; **62**: 1764–71

80. Cape E, Hall RJ, van Munster BC, et al. Cerebrospinal fluid markers of neuroinflammation in delirium: a role for interleukin-1β in delirium after hip fracture. *J Psychosom Res*; 2014; **77**: 219–25

81. Seo JS, Park SW, Lee YS, Chung C, Kim YB. Risk factors for delirium after spine surgery in elderly patients. *J Korean Neurosurg Soc*; 2014; **56**: 28–33

82. M.m. De Castro S, Ünlü Ç, B. Tuynman J, et al. Incidence and risk factors of delirium in the elderly general surgical patient. *Am J Surg*; 2014; **208**: 26–32

83. Fan Y-X, Liu F-F, Jia M, et al. Comparison of restrictive and liberal transfusion strategy on postoperative delirium in aged patients following total hip replacement: a preliminary study. *Arch Gerontol Geriatr*; 2014; **59**: 181–5

84. Wu Y, Wang J, Wu A, Yue Y. Do fluctuations in endogenous melatonin levels predict the occurrence of postoperative cognitive dysfunction (POCD)? *Int J Neurosci*; 2014; **124**: 787–91

85. Deiner S, Lin H-M, Bodansky D, Silverstein J, Sano M. Do stress markers and anesthetic technique predict delirium in the elderly? *Dement Geriatr Cogn Disord*; 2014; **38**: 366-374

86. de Jonghe A, van Munster BC, Goslings JC, et al. Effect of melatonin on incidence of delirium among patients with hip fracture: a multicentre, double-blind randomized controlled trial. *Can Med Assoc J*; 2014; **186**: E547–56

87. Saporito A, Sturini E. Incidence of postoperative delirium is high even in a population without known risk factors. *J Anesth*; 2014; **28**: 198–201

88. Flikweert ER, Izaks GJ, Knobben BA, Stevens M, Wendt K. The development of a comprehensive multidisciplinary care pathway for patients with a hip fracture: design and results of a clinical trial. *BMC Musculoskelet Disord*; 2014; **15**: 188

89. Anbar R, Beloosesky Y, Cohen J, et al. Tight calorie control in geriatric patients following hip fracture decreases complications: a randomized, controlled study. *Clin Nutr*; 2014; **33**: 23–8

90. Wang N-Y, Hirao A, Sieber F. Association between intraoperative blood pressure and postoperative delirium in elderly hip fracture patients. *PLoS One*; 2015; **10**

91. Yang X, Li Z, Gao C, Liu R. Effect of dexmedetomidine on preventing agitation and delirium after microvascular free flap surgery: a randomized, double-blind, control study. *J oral Maxillofac Surg*; 2015; **73**: 1065–72

92. Hempenius L, Slaets JPJ, van Asselt DZB, et al. Interventions to prevent postoperative delirium in elderly cancer patients should be targeted at those undergoing nonsuperficial surgery with special attention to the cognitive impaired patients. *Eur J Surg Oncol*; 2015; **41**: 28–33

93. Chen Y-L, Lin H-C, Lin K-H, et al. Low hemoglobin level is associated with the development of delirium after hepatectomy for hepatocellular carcinoma patients. *PLoS One*; 2015; **10**

94. Chevillon C, Hellyar M, Madani C, Kerr K, Kim SC. Preoperative education on postoperative delirium, anxiety, and knowledge in pulmonary thrombo-endarterectomy patients. *Am J Crit CARE*; 2015; **24**: 164–71

95. Tai S, Xu L, Zhang L, Fan S, Liang C. Preoperative risk factors of postoperative delirium after transurethral prostatectomy for benign prostatic hyperplasia. *Int J Clin Exp Med*; 2015; **8**: 4569–74

96. Gottschalk A, Hubbs J, Vikani AR, Gottschalk LB, Sieber FE. The impact of incident postoperative delirium on survival of elderly patients after surgery for hip fracture repair. *Anesth Analg;* 2015; **121**: 1336–43

97. Wang R, Chen J, Wu G. Variable lung protective mechanical ventilation decreases incidence of postoperative delirium and cognitive dysfunction during open abdominal surgery. *Int J Clin Exp Med*; 2015; **8**: 21208-21214

98. Raats JW, Van Eijsden WA, Crolla RMPH, Steyerberg EW, Van Der Laan L. Risk factors and outcomes for postoperative delirium after major surgery in elderly patients. *PLoS One*; 2015; **10**

99. Kratz T, Heinrich M, Schlauß E, Diefenbacher A. The preventing of postoperative delirium. *Dtsch Aerzteblatt Online*; 2015; **112**

100. Liang CK, Chu CL, Chou MY, et al. Developing a prediction model for post-operative delirium and long-term outcomes among older patients receiving elective orthopedic surgery: a prospective cohort study in Taiwan. *Rejuvenation Res*; 2015; **18**: 347–55

101. Nie H, Yang Y-X, Wang Y, Liu Y, Zhao B, Luan B. Effects of continuous fascia iliaca compartment blocks for postoperative analgesia in patients with hip fracture. *Pain Res Manag;* 2015; **20**: 210–2

102. Schmidt M, Eckardt R, Scholtzl K, et al. Patient empowerment improved perioperative quality of care in cancer patients aged >= 65 years a randomized controlled trial. *PLoS One;* 2015; **10**

103. Punjasawadwong Y, Pipanmekaporn T, Wongpakaran N. Optimized anesthesia to reduce incidence of postoperative delirium in elderly undergoing elective, non-cardiac surgery: a randomized controlled trial. *Anesth Analg;* 2016; **123**: 211

104. Hall RJ, Watne LO, Idland A-V, et al. Cerebrospinal fluid levels of neopterin are elevated in delirium after hip fracture. *J Neuroinflammation;* 2016; **13**: 170

105. Maekawa Y, Sugimoto K, Yamasaki M, et al. Comprehensive Geriatric Assessment is a useful predictive tool for postoperative delirium after gastrointestinal surgery in old-old adults. *Geriatr Gerontol Int*; 2016; **16**: 1036–42

106. Mohammadi M, Ahmadi M, Khalili H, Cheraghchi H, Arbabi M. Cyproheptadine for the prevention of postoperative delirium: a pilot study. *Ann Pharmacother*; 2016; **50**: 180–7

107. Jeong DM, Kim JA, Ahn HJ, Yang M, Heo BY, Lee SH. Decreased incidence of postoperative delirium in robot-assisted thoracoscopic esophagectomy compared with open transthoracic esophagectomy. *Surg Laparosc Endosc Percutaneous Tech;* 2016; **26**: 516–22

108. Brown CH 4th CH, LaFlam A, Max L, et al. Delirium after spine surgery in older adults: incidence, risk factors, and outcomes. *J Am Geriatr Soc;* 2016; **64**: 2101–8

109. Kim MY, Park UJ, Kim HT, Cho WH. DELirium Prediction Based on Hospital Information (Delphi) in general surgery patients. *Medicine (Baltimore);* 2016; **95**: e3072

110. Su X, Meng Z-T, Wu X-H, et al. Dexmedetomidine for prevention of delirium in elderly patients after non-cardiac surgery: a randomised, double-blind, placebo-controlled trial. *Lancet*; 2016; **388**: 1893–902

111. Liu Y, Ma L, Gao M, Guo W, Ma Y. Dexmedetomidine reduces postoperative delirium after joint replacement in elderly patients with mild cognitive impairment. *Aging Clin Exp Res*; 2016; **28**: 729–36

112. Girard N. Evidence appraisal of Winter A, Steurer MP, Dullenkopf A. Postoperative delirium assessed by post anesthesia care unit staff utilizing the Nursing Delirium Screening Scale: a prospective observational study of 1000 patients in a single Swiss institution.: BMC Anesthesiol. 2015;15:184. *AORN J*; 2016; **104**: 364–9

113. Moerman S, Vochteloo AJH, Tuinebreijer WE, Maier AB, Mathijssen NMC, Nelissen RGHH. Factors associated with the course of health-related quality of life after a hip fracture. *Arch Orthop Trauma Surg*; 2016; **136**: 935–43

114. Stukenberg S, Franck M, Spies CD, Neuner B, Myers I, Radtke FM. How can postoperative delirium be predicted in advance? A secondary analysis comparing three methods of early assessment in elderly patients. *Minerva Anestesiol*; 2016; **82**: 751–9

115. Zhang Z-Y, Gao D-P, Yang J-J, et al. Impact of length of red blood cells transfusion on postoperative delirium in elderly patients undergoing hip fracture surgery: a cohort study. *Injury*; 2016; **47**: 408–12

116. Guo Y, Sun L, Li L, et al. Impact of multicomponent, nonpharmacologic interventions on perioperative cortisol and melatonin levels and postoperative delirium in elderly oral cancer patients. *Arch Gerontol Geriatr*; 2016; **62**: 112–7

117. Watne LO, Idland A-V, Fekkes D, et al. Increased CSF levels of aromatic amino acids in hip fracture patients with delirium suggests higher monoaminergic activity. *BMC Geriatr;* 2016; **16**

118. Van Grootven B, Detroyer E, Devriendt E, et al. Is preoperative state anxiety a risk factor for postoperative delirium among elderly hip fracture patients? *Geriatr Gerontol Int*; 2016; **16**: 948-955

119. Hempenius L, Slaets JPJ, van Asselt D, de Bock TH, Wiggers T, van Leeuwen BL. Long term outcomes of a geriatric liaison intervention in frail elderly cancer patients. *PLoS One;* 2016; **11**: e0143364

120. Franck M, Nerlich K, Neuner B, et al. No convincing association between post-operative delirium and post-operative cognitive dysfunction: a secondary analysis. *Acta Anaesthesiol Scand*; 2016; **60**: 1404–14

121. Pinho C, Cruz S, Santos A, Abelha FJ. Postoperative delirium: age and low functional reserve as independent risk factors. *J Clin Anesth;* 2016; **33**: 507–13

122. Zheng Y-B, Ruan G-M, Fu J-X, Su Z-L, Cheng P, Lu J-Z. Postoperative plasma 8-iso-prostaglandin F2α levels are associated with delirium and cognitive dysfunction in elderly patients after hip fracture surgery. *Clin Chim Acta*; 2016; **455**: 149–53

123. Sun L, Jia P, Zhang J, et al. Production of inflammatory cytokines, cortisol, and Abeta1-40 in elderly oral cancer patients with postoperative delirium. *Neuropsychiatr Dis Treat*; 2016; **12 CC**-: 2789-2795

124. van der Zanden V, Beishuizen SJSJSJ, Scholtens RMRM, de Jonghe A, de Rooij SESE, van Munster BCBCBC. The effects of blood transfusion on delirium incidence. *J Am Med Dir Assoc*; 2016; **17**: 748–53

125. Neuman MD, Mehta S, Bannister ER, Hesketh PJ, Horan AD, Elkassabany NM. Pilot randomized controlled trial of spinal versus general anesthesia for hip fracture surgery. *J Am Geriatr Soc*; 2016; **64**: 2604-2606

126. Tei M, Wakasugi M, Kishi K, Tanemura M, Akamatsu H. Incidence and risk factors of postoperative delirium in elderly patients who underwent laparoscopic surgery for colorectal cancer. *Int J Colorectal Dis*; 2016; **31**: 67–73

127. Wu XH, Cui F, Zhang C, et al. Low-dose dexmedetomidine improves sleep quality pattern in elderly patients after noncardiac surgery in the intensive care unit: a pilot randomized controlled trial. *Anesthesiology*; 2016; **125**: 979–91

128. Chu CS, Liang CK, Chou MY, et al. Short-Form Mini Nutritional Assessment as a useful method of predicting the development of postoperative delirium in elderly patients undergoing orthopedic surgery. *Gen Hosp Psychiatry*; 2016; **38**: 15–20

129. Neerland BE, Hall RJ, Seljeflot I, et al. Associations between delirium and preoperative cerebrospinal fluid C-reactive protein, interleukin-6, and interleukin-6 receptor in individuals with acute hip fracture. *J Am Geriatr Soc*; 2016; **64**: 1456–63

130. Yen TE, Allen JC, Rivelli SK, et al. Association between serum IGF-I levels and postoperative delirium in elderly subjects undergoing elective knee arthroplasty. *Sci Rep*; 2016; **6**

131. Guo Y, Jia P, Zhang J, Wang X, Jiang H, Jiang W. Prevalence and risk factors of postoperative delirium in elderly hip fracture patients. *J Int Med Res*; 2016; **44**: 317–27

132. Xue P, Wu Z, Wang K, Tu C, Wang X. Incidence and risk factors of postoperative delirium in elderly patients undergoing transurethral resection of prostate: a prospective cohort study. *Neuropsychiatr Dis Treat*; 2016; **12**: 137–42

133. Sato T, Hatakeyama S, Okamoto T, et al. Slow gait speed and rapid renal function decline are risk factors for postoperative delirium after urological surgery. *PLoS One*; 2016; **11**

134. Sugano N, Aoyama T, Sato T, et al. Randomized phase II study of TJ-54 (Yokukansan) for postoperative delirium in gastrointestinal and lung malignancy patients. *Ann Oncol*; 2017; **28**: x159

135. Youn YC, Shin H-W, Choi B-S, Kim S, Lee J-Y, Ha Y-C. Rivastigmine patch reduces the incidence of postoperative delirium in older patients with cognitive impairment. *Int J Geriatr Psychiatry*; 2017; **32**: 1079–84

136. El-Gabalawy R, Patel R, Kilborn K, et al. A novel stress-diathesis model to predict risk of post-operative delirium: implications for intra-operative management. *Front Aging Neurosci*; 2017; **9**

137. Ha A, Krasnow R, Hsieh T, et al. A predictive risk stratification model for delirium after major urologic cancer surgeries. *J Urol*; 2017; **197**: e1157

138. Nadler JW, Evans JL, Fang E, et al. A randomised trial of peri-operative positive airway pressure for postoperative delirium in patients at risk for obstructive sleep apnoea after regional anaesthesia with sedation or general anaesthesia for joint arthroplasty. *Anaesthesia*; 2017; **72**: 729–36

139. Mazzola P, Ward L, Zazzetta S, et al. Association between preoperative malnutrition and postoperative delirium after hip fracture surgery in older adults. *J Am Geriatr Soc*; 2017; **65**: 1222–8

140. Dong-Nan Yu. Comparison of post-anesthesia delirium in elderly patients treated with dexmedetomidine and midazolam maleate after thoracic surgery. *Biomed Res*; 2017; **28**: 6852-6855

141. Petersen PB, Jørgensen CC, Kehlet H. Delirium after fast-track hip and knee arthroplasty - a cohort study of 6331 elderly patients. *Acta Anaesthesiol Scand*; 2017; **61**: 767–72

142. Su X, Meng ZT, Wu XH. Dexmedetomidine for prevention of delirium in elderly patients after noncardiac surgery: a randomised, double-blind, placebo-controlled trial. *J Neurosurg Anesthesiol;* 2017; **29**: 178-179

143. Inoue R, Sumitani M, Ogata T, et al. Direct evidence of central nervous system axonal damage in patients with postoperative delirium: a preliminary study of pNF-H as a promising serum biomarker. *Neurosci Lett*; 2017; **653**: 39–44

144. Chen CC-H, Li H-C, Liang J-T, et al. Effect of a modified hospital elder life program on delirium and length of hospital stay in patients undergoing abdominal surgery: a cluster randomized clinical trial. *JAMA Surg;* 2017; **152**: 827–34

145. Fukata S, Kawabata Y, Fujishiro K, et al. Haloperidol prophylaxis for preventing aggravation of postoperative delirium in elderly patients: a randomized, open-label prospective trial. *Surg Today*; 2017; **47**: 815–26

146. Vasunilashorn SM, Dillon ST, Inouye SK, et al. High C-reactive protein predicts delirium incidence, duration, and feature severity after major noncardiac surgery. *J Am Geriatr Soc;* 2017; **65**: e109–16

147. Xin X, Xin F, Chen X, et al. Hypertonic saline for prevention of delirium in geriatric patients who underwent hip surgery. *J Neuroinflammation;* 2017; **14**: 221

148. Deiner S, Luo X, Lin H-M, et al. Intraoperative infusion of dexmedetomidine for prevention of postoperative delirium and cognitive dysfunction in elderly patients undergoing major elective noncardiac surgery: a randomized clinical trial. *JAMA Surg;* 2017; **152**: e171505

149. Dong R, Sun L, Lu Y, Yang X, Peng M, Zhang Z. NeurimmiRs and postoperative delirium in elderly patients undergoing total hip/knee replacement: a pilot study. *Front Aging Neurosci*; 2017; **9**

150. Cunningham EL, Mawhinney T, Beverland D, et al. Observational cohort study examining apolipoprotein E status and preoperative neuropsychological performance as predictors of post-operative delirium in an older elective arthroplasty population. *Age Ageing*; 2017; **46**: 779–86

151. Mu D-LD-LD-L, Zhang D-Z, Wang D-XD-XD-X, et al. Parecoxib supplementation to morphine analgesia decreases incidence of delirium in elderly patients after hip or knee replacement surgery: a randomized controlled trial. *Anesth Analg*; 2017; **124**: 1992–2000

152. Leung JM, Sands LP, Chen N, et al. Perioperative gabapentin does not reduce postoperative delirium in older surgical patients: a randomized clinical trial. *Anesthesiology;* 2017; **127**: 633–44

153. Culley DJ, Flaherty D, Fahey MC, et al. Poor performance on a preoperative cognitive screening test predicts postoperative complications in older orthopedic surgical patients. *Anesthesiology;* 2017; **127**: 765–74

154. Bhattacharya B, Maung A, Barre K, et al. Postoperative delirium is associated with increased intensive care unit and hospital length of stays after liver transplantation. *J Surg Res;* 2017; **207**: 223–8

155. Wu X-M, Xu W-C, Yu Y-J, Han L, Zhang J, Yang L-J. Postoperative serum thioredoxin concentrations correlate with delirium and cognitive dysfunction after hip fracture surgery in elderly patients. *Clin Chim Acta*; 2017; **466**: 93–7

156. Todd OM, Gelrich L, MacLullich AM, Driessen M, Thomas C, Kreisel SH. Sleep disruption at home as an independent risk factor for postoperative delirium. *J Am Geriatr Soc*; 2017; **65**: 949–57

157. Tanaka P, Goodman S, Sommer BR, Maloney W, Huddleston J, Lemmens HJ. The effect of desflurane versus propofol anesthesia on postoperative delirium in elderly obese patients undergoing total knee replacement: a randomized, controlled, double-blinded clinical trial. *J Clin Anesth*; 2017; **39**: 17–22

158. Radovanović D, Radovanović Z, Škorić-Jokić S, et al. Thoracic epidural versus intravenous patient-controlled analgesia after open colorectal cancer surgery. *ACTA Clin Croat*; 2017; **56**: 244–54

159. Freter S, Koller K, Dunbar M, MacKnight C, Rockwood K. Translating delirium prevention strategies for elderly adults with hip fracture into routine clinical care: a pragmatic clinical trial. *J Am Geriatr Soc*; 2017; **65**: 567–73

160. Beishuizen SJE, Scholtens RM, van Munster BC, de Rooij SE. Unraveling the relationship between delirium, brain damage, and subsequent cognitive decline in a cohort of individuals undergoing surgery for hip fracture. *J Am Geriatr Soc*; 2017; **65**: 130–6

161. Elsamadicy AA, Adogwa O, Lydon E, et al. Depression as an independent predictor of postoperative delirium in spine deformity patients undergoing elective spine surgery. *J Neurosurg Spine*; 2017; **27**: 209–14

162. van der Sluis FJ, Buisman PL, Meerdink M, et al. Risk factors for postoperative delirium after colorectal operation. *Surg (United States)*; 2017; **161**: 704–11

163. Xiang D, Xing H, Tai H, Xie G. Preoperative C-reactive protein as a risk factor for postoperative delirium in elderly patients undergoing laparoscopic surgery for colon carcinoma. *Biomed Res Int*; 2017; **2017**

164. Wang L, Seok S, Kim S, Kim K, Lee S, Lee K. The risk factors of postoperative delirium after total knee arthroplasty. *J Knee Surg*; 2017; **30**: 600–5

165. Huang J, Razak HRBA, Yeo SJ. Incidence of postoperative delirium in patients undergoing total knee arthroplasty-an Asian perspective. *Ann Transl Med*; 2017; **5**

166. Hshieh TT, Saczynski J, Gou RY, et al. Trajectory of functional recovery after postoperative delirium in elective surgery. *Ann Surg;* 2017; **265**: 647–53

167. Zhou Y, Li Y, Wang K. Bispectral index monitoring during anesthesia promotes early postoperative recovery of cognitive function and reduces acute delirium in elderly patients with colon carcinoma: a prospective controlled study using the attention network test. *Med Sci Monit*; 2018; **24**: 7785–93

168. Racine AM, Fong TG, Gou Y, et al. Clinical outcomes in older surgical patients with mild cognitive impairment. *Alzheimers Dement;* 2018; **14**: 590–600

169. Hassel B, Mariussen E, Idland A-V, et al. CSF sodium at toxic levels precedes delirium in hip fracture patients. *Neurotoxicology*; 2018; **69**: 11–6

170. Unneby A, Svensson O, Lindgren B-M, Bergström U, Gustafson Y. Delirium and other complications during hospital stay related to femoral nerve block vs conventional pain management among patients with hip fracture: a randomised controlled trial. *Eur Geriatr Med*; 2018; **9**: S30

171. Sieber F, Neufeld K, Gottschalk A, et al. Depth of sedation as an interventional target to reduce postoperative delirium: a randomized clinical trial. *Anesth Analg*; 2018; **126**: 282

172. Clemmesen CG, Lunn TH, Kristensen MT, Palm H, Foss NB. Effect of a single pre-operative 125 mg dose of methylprednisolone on postoperative delirium in hip fracture patients; a randomised, double-blind, placebo-controlled trial. *Anaesthesia*; 2018; **73**: 1353–60

173. Xuan Y, Fan R, Chen J, et al. Effects of dexmedetomidine for postoperative delirium after joint replacement in elderly patients: a randomized, double-blind, and placebo-controlled trial. *Int J Clin Exp Med*; 2018; **11**: 13147–57

174. Lee H, Ju J-W, Oh S-Y, Kim J, Jung CW, Ryu HG. Impact of timing and duration of postoperative delirium: a retrospective observational study. *Surgery;* 2018; **164**: 137–43

175. Hight DF, Sleigh J, Winders JD, et al. Inattentive delirium vs. disorganized thinking: a new axis to subcategorize PACU delirium. *Front Syst Neurosci;* 2018; **12**

176. McDonald SR, Heflin MT, Whitson HE, et al. Association of integrated care coordination with postsurgical outcomes in high-risk older adults the Perioperative Optimization of Senior Health (POSH) initiative. *JAMA Surg*; 2018; **153**: 454–62

177. Munjupong S, Sripon T, Siripoonyothai S, Jesadapatarakul N, Poojinya T, Chernsirikasem N. Incidence and risk factors of emergence delirium after general and regional anesthesia in elective non-cardiac surgery patients. *J Med Assoc Thail*; 2018; **101**: 1653–8

178. Mosk CA, van Vugt JLA, de Jonge H, et al. Low skeletal muscle mass as a risk factor for postoperative delirium in elderly patients undergoing colorectal cancer surgery. *Clin Interv Aging*; 2018; **13**: 2097–106

179. Fuchita M, Perkins A, Khan S, Kesler K, Khan B. Perioperative risk factors for postoperative delirium in patients undergoing esophagectomy. *Crit Care Med*; 2018; **46**: 369

180. Khan BA, Perkins AJ, Campbell NL, et al. Preventing postoperative delirium after major noncardiac thoracic surgery-a randomized clinical trial. *J Am Geriatr Soc*; 2018; **66**: 2289–97

181. Tahir M, Malik SS, Ahmed U, Kozdryk J, Naqvi SH, Malik A. Risk factors for onset of delirium after neck of femur fracture surgery: a prospective observational study. *SICOT-J;* 2018; **4**

182. Lee C, Lee CH, Lee G, Lee M, Hwang J. The effect of the timing and dose of dexmedetomidine on postoperative delirium in elderly patients after laparoscopic major non-cardiac surgery: a double blind randomized controlled study. *J Clin Anesth*; 2018; **47**: 27–32

183. Arnold GM, Coburn M, Rossaint R, et al. The hip fracture surgery in elderly patients (HIPELD) study to evaluate xenon anaesthesia for the prevention of postoperative delirium: a multicentre, randomized clinical trial. *Br J Anaesth*; 2018; **120**: 127-137

184. Oh TK, Park HY, Shin H-J, Jeon Y-T, Do S-H, Hwang J-W. The role of perioperative statin use in the prevention of delirium after total knee replacement under spinal anesthesia. *J Arthroplasty*; 2018; **33**: 3666-3671.e1

185. Gai N, Lavi R, Jones PMPMPM, Lee H, Naudie D, Bainbridge D. The use of point-of-care ultrasound to diagnose patent foramen ovale in elective hip and knee arthroplasty patients and its association with postoperative delirium. *Can J Anaesth*; 2018; **65**: 619–26

186. Adogwa O, Elsamadicy AA, Vuong VD, et al. Association between baseline cognitive impairment and postoperative delirium in elderly patients undergoing surgery for adult spinal deformity. *J Neurosurg Spine*; 2018; **28**: 103–8

187. Kim KH, Kang SY, Shin DA, et al. Parkinson’s disease-related non-motor features as risk factors for post-operative delirium in spinal surgery. *PLoS One*; 2018; **13**

188. Xie S, Xie M. Effect of dexmedetomidine on postoperative delirium in elderly patients undergoing hip fracture surgery. Pak J Pharm Sci; 2018; 31(5(Special)): 2277-2281.

189. Hong N, Park JY. The motoric types of delirium and estimated blood loss during perioperative period in orthopedic elderly patients. *Biomed Res Int*; 2018; **2018**

190. Monacelli F, Signori A, Prefumo M, et al. Delirium, frailty, and fast-track surgery in oncogeriatrics: Is there a link? *Dement Geriatr Cogn Dis Extra*; 2018; **8**: 33–41

191. Morino T, Hino M, Yamaoka S, et al. Risk factors for delirium after spine surgery: an age-matched analysis. *Asian Spine J*; 2018; **12**: 703–9

192. Coburn M, Sanders RD, Maze M, et al. The hip fracture surgery in elderly patients (HIPELD) study to evaluate xenon anaesthesia for the prevention of postoperative delirium: a multicentre, randomized clinical trial. *Br J Anaesth*; 2018; **120**: 127–37

193. Bellelli G, Carnevali L, Corsi M, et al. The impact of psychomotor subtypes and duration of delirium on 6-month mortality in hip-fractured elderly patients. *Int J Geriatr Psychiatry*; 2018; **33**: 1229–35

194. Tzimas P, Samara E, Petrou A, Korompilias A, Chalkias A, Papadopoulos G. The influence of anesthetic techniques on postoperative cognitive function in elderly patients undergoing hip fracture surgery: general vs spinal anesthesia. *Injury*; 2018; **49**: 2221–6

195. Gao F, Zhang Q, Li Y, et al. Transcutaneous electrical acupoint stimulation for prevention of postoperative delirium in geriatric patients with silent lacunar infarction: a preliminary study. *Clin Interv Aging;* 2018; **13**: 2127–34

196. Lachmann G, Kant I, Lammers F, et al. Cerebral microbleeds are not associated with postoperative delirium and postoperative cognitive dysfunction in older individuals. *PLoS One;* 2019; **14**

197. Cunningham EL, McGuinness B, McAuley DF, et al. CSF beta-amyloid 1-42 concentration predicts delirium following elective arthroplasty surgery in an observational cohort study. *Ann Surg*; 2019; **269**: 1200–5

198. Choi H, Shin B, Yoo H, et al. Early corticosteroid treatment for postoperative acute lung injury after lung cancer surgery. *Ther Adv Respir Dis*; 2019; **13**

199. Chen K, Wang L, Wang Q, et al. Effects of pneumoperitoneum and steep Trendelenburg position on cerebral hemodynamics during robotic-assisted laparoscopic radical prostatectomy: a randomized controlled study. *Medicine (Baltimore);* 2019; **98**: e15794

200. Vlisides PE, Das AR, Thompson AM, et al. Home-based cognitive prehabilitation in older surgical patients: a feasibility study. *J Neurosurg Anesthesiol;* 2019; **31**: 212–7

201. Windmann V, Spies C, Knaak C, et al. Intraoperative hyperglycemia increases the incidence of postoperative delirium. *Minerva Anestesiol*; 2019; **85**: 1201–10

202. Langer T, Santini A, Zadek F, et al. Intraoperative hypotension is not associated with postoperative cognitive dysfunction in elderly patients undergoing general anesthesia for surgery: results of a randomized controlled pilot trial. *J Clin Anesth;* 2019; **52**: 111–8

203. Gutierrez R, Egaña JI, Saez I, et al. Intraoperative low alpha power in the electroencephalogram is associated with postoperative subsyndromal delirium. *Front Syst Neurosci;* 2019; **13**

204. Kim JA, Ahn HJ, Yang M, Lee SH, Jeong H, Seong BG. Intraoperative use of dexmedetomidine for the prevention of emergence agitation and postoperative delirium in thoracic surgery: a randomized-controlled trial. *Can J Anaesth*; 2019; **66**: 371–9

205. Zhao B, Ni Y, Tian X. Low plasma cholinesterase activity is associated with postoperative delirium after noncardiac surgery in elderly patients: a prospective observational study. *Psychosomatics*; 2019; **60**: 190–6

206. Vlisides PE, Thompson A, Kunkler BS, Maybrier HR, Avidan MS, Mashour GA. Perioperative epidural use and risk of delirium in surgical patients: a secondary analysis of the PODCAST trial. *Anesth Analg*; 2019; **128**: 944–52

207. Schmid S, Blobner M, Haas B, et al. Perioperative multi-system optimization protocol in elderly hip fracture patients: a randomized-controlled trial. *Can J Anesth*; 2019; **66**: 1472–82

208. Fuchita M, Khan SH, Perkins AJ, et al. Perioperative risk factors for postoperative delirium in patients undergoing esophagectomy. *Ann Thorac Surg*; 2019; **108**: 190–5

209. Chaiwat O, Chanidnuan M, Pancharoen W, et al. Postoperative delirium in critically ill surgical patients: incidence, risk factors, and predictive scores. *BMC Anesthesiol;* 2019; **19**: 39

210. Park EA, Kim MY. Postoperative delirium is associated with negative outcomes and long-term mortality in elderly Koreans: a retrospective observational study. *Medicina (Kaunas);* 2019; **55**

211. Tao L, Xiaodong X, Qiang M, Jiao L, Xu Z. Prediction of postoperative delirium by comprehensive geriatric assessment among elderly patients with hip fracture. *Ir J Med Sci*; 2019; **188**: 1311–5

212. Oe S, Togawa D, Yamato Y, et al. Preoperative age and prognostic nutritional index are useful factors for evaluating postoperative delirium among patients with adult spinal deformity. *Spine (Phila Pa 1976)*; 2019; **44**: 472–8

213. Wada S, Inoguchi H, Sadahiro R, et al. Preoperative anxiety as a predictor of delirium in cancer patients: a prospective observational cohort study. *World J Surg*; 2019; **43**: 134–42

214. Peng J, Wu G, Chen J, Chen H. Preoperative C-reactive protein/albumin ratio, a risk factor for postoperative delirium in elderly patients after total joint arthroplasty. *J Arthroplasty*; 2019; **34**: 2601–5

215. Wang X, Feng K, Liu H, et al. Regional cerebral oxygen saturation and postoperative delirium in endovascular surgery: a prospective cohort study. *Trials;* 2019; **20**: 504

216. Li Q-H, Yu L, Yu Z-W, et al. Relation of postoperative serum S100A12 levels to delirium and cognitive dysfunction occurring after hip fracture surgery in elderly patients. *Brain Behav;* 2019; **9**: e01176

217. Wang Y, Yu H, Qiao H, Li C, Chen K, Shen X. Risk factors and incidence of postoperative delirium in patients undergoing laryngectomy. *Otolaryngol neck Surg*; 2019; **161**: 807–13

218. Xu W-B, Hu Q-H, Wu C-N, Fan Z-K, Song Z-F. Serum soluble fibrinogen-like protein 2 concentration predicts delirium after acute pancreatitis. *BRAIN Behav;* 2019; **9**

219. Roijers JP, Hopmans CJ, Janssen TL, et al. The role of delirium and other risk factors on mortality in elderly patients with critical limb ischemia undergoing major lower limb amputation. *Ann Vasc Surg*; 2019; **60**: 270-278.e2

220. Shim E-J, Noh HL, Lee K-M, et al. Trajectory of severity of postoperative delirium symptoms and its prospective association with cognitive function in patients with gastric cancer: results from a prospective observational study. *Support care cancer*; 2019; **27**: 2999–3006

221. Pan Z, Huang K, Huang W, et al. The risk factors associated with delirium after lumbar spine surgery in elderly patients. *Quant Imaging Med Surg*; 2019; **9**: 700–10

222. Susano MJ, Scheetz SD, Grasfield RH, et al. Retrospective analysis of perioperative variables associated with postoperative delirium and other adverse outcomes in older patients after spine surgery. *J Neurosurg Anesthesiol*; 2019; p. 385–91

223. Kin K, Yasuhara T, Tomita Y, Umakoshi M, Morimoto J, Date I. SF-36 scores predict postoperative delirium after surgery for cervical spondylotic myelopathy. *J Neurosurg Spine*; 2019; **30**: 777–82

224. Guo Y, Li Y, Zhang Y, et al. Post-operative delirium associated with metabolic alterations following hemi-arthroplasty in older patients. *Age Ageing*; 2019; **49**: 88–95

225. de Jong L, van Rijckevorsel VAJIM, Raats JW, Klem TMAL, Kuijper TM, Roukema GR. Delirium after hip hemiarthroplasty for proximal femoral fractures in elderly patients: risk factors and clinical outcomes. *Clin Interv Aging*; 2019; **14**: 427–35

226. Clemmesen CG, Palm H, Foss NB. Delay in detection and treatment of perioperative anemia in hip fracture surgery and its impact on postoperative outcomes. *Injury*; 2019; **50**: 2034–9

227. Mueller A, Spies CD, Eckardt R, et al. Anticholinergic burden of long-term medication is an independent risk factor for the development of postoperative delirium: a clinical trial. *J Clin Anesth;* 2020; **61**

228. Maheshwari K, Ahuja S, Khanna AK, et al. Association between perioperative hypotension and delirium in postoperative critically ill patients: a retrospective cohort analysis. *Anesth Analg*; 2020; **130**: 636–43

229. Farag E, Liang C, Masha EJ, et al. Association between use of angiotensin-converting enzyme inhibitors or angiotensin receptor blockers and postoperative delirium. *Anesthesiology*; 2020; 119–32

230. Unneby A, Svensson PO, Gustafson PY, et al. Complications with focus on delirium during hospital stay related to femoral nerve block compared to conventional pain management among patients with hip fracture - a randomised controlled trial. *Injury;* 2020; **51**: 1634–41

231. Zhong X, Lin J-Y, Li L, Barrett AM, Poeran J, Mazumdar M. Derivation and validation of a novel comorbidity-based delirium risk index to predict postoperative delirium using national administrative healthcare database. *Health Serv Res*; 2020

232. Shi H, Du X, Wu F, Hu Y, Xv Z, Mi W. Dexmedetomidine improves early postoperative neurocognitive disorder in elderly male patients undergoing thoracoscopic lobectomy. *Exp Ther Med*; 2020; **20**: 3867–76

233. Bielza R, Llorente J, Thuissard IJ, et al. Effect of intravenous iron on functional outcomes in hip fracture: a randomised controlled trial. *Age Ageing;* 2021; **50**: 127–34

234. Lee H, Yang SM, Chung J, et al. Effect of perioperative low-dose dexmedetomidine on postoperative delirium after living-donor liver transplantation: a randomized controlled trial. *Transplant Proc*; 2020; **52**: 239–45

235. Zhang W, Wang T, Wang G, Yang M, Zhou Y, Yuan Y. Effects of dexmedetomidine on postoperative delirium and expression of IL-1β, IL-6, and TNF-α in elderly patients after hip fracture operation. *Front Pharmacol*; 2020; **11**

236. Xu X, Hu X, Wu Y, et al. Effects of different BP management strategies on postoperative delirium in elderly patients undergoing hip replacement: a single center randomized controlled trial. *J Clin Anesth;* 2020; **62**

237. Zhang Y, He S-T, Nie B, Li X-Y, Wang D-X. Emergence delirium is associated with increased postoperative delirium in elderly: a prospective observational study. *J Anesth;* 2020; **34**: 675–87

238. Yuan Y, Li Z, Yang N, et al. Exosome α-synuclein release in plasma may be associated with postoperative delirium in hip fracture patients. *Front Aging Neurosci*; 2020; **12**

239. Ortner F, Eberl M, Otto S, et al. Patient-related and anesthesia-dependent determinants for postoperative delirium after oral and maxillofacial surgery. Results from a register-based case-control study. *J Stomatol oral Maxillofac Surg*; 2020

240. Hindiskere S, Kim H-S, Han I. Postoperative delirium in patients undergoing surgery for bone metastases. *Medicine (Baltimore);* 2020; **99**: e20159

241. Onuma H, Inose H, Yoshii T, et al. Preoperative risk factors for delirium in patients aged ≥75 years undergoing spinal surgery: a retrospective study. *J Int Med Res*; 2020; **48**

242. Knaak C, Brockhaus W-R, Spies C, et al. Presurgical cognitive impairment is associated with postoperative delirium and postoperative cognitive dysfunction. *Minerva Anestesiol*; 2020; **86**: 394–403

243. Shen Q-H, Li H-F, Zhou X-Y, Lu Y-P, Yuan X-Z. Relation of serum melatonin levels to postoperative delirium in older patients undergoing major abdominal surgery. *J Int Med Res*; 2020; **48**

244. Mei B, Xu G, Han W, et al. The benefit of dexmedetomidine on postoperative cognitive function is unrelated to the modulation on peripheral inflammation: a single-center, prospective, randomized study. *Clin J Pain*; 2020; **36**: 88–95

245. Chuan A, Zhao L, Tillekeratne N, et al. The effect of a multidisciplinary care bundle on the incidence of delirium after hip fracture surgery: a quality improvement study. *Anaesthesia*; 2020; **75**: 63–71

246. Uysal Aİ, Altıparmak B, Yaşar E, et al. The effects of early femoral nerve block intervention on preoperative pain management and incidence of postoperative delirium geriatric patients undergoing trochanteric femur fracture surgery: a randomized controlled trial. *Ulus travma ve acil cerrahi Derg = Turkish J trauma Emerg Surg*; 2020; **26**: 109–14

247. Mei X, Zheng H-L, Li C, et al. The effects of propofol and sevoflurane on postoperative delirium in older patients: a randomized clinical trial study. *J Alzheimer’s Dis* China; 2020; **76**: 1627–36

248. Wang J, Zhu L, Li Y, Yin C, Hou Z, Wang Q. The potential role of lung-protective ventilation in preventing postoperative delirium in elderly patients undergoing prone spinal surgery: a preliminary study. *Med Sci Monit*; 2020; **26**

249. Mungan I, Türksal E, Sari S, Bostanci E, Turan S. The relationship between postoperative outcomes and delirium after liver transplantation in intensive care unit: a single-center experience. *Indian J Transplant* Turkey; 2020; **14**: 136–40

250. Moslemi R, Khalili H, Mohammadi M, Mehrabi Z, Mohebbi N. Thiamine for prevention of postoperative delirium in patients undergoing gastrointestinal surgery: a randomized clinical trial. *J Res Pharm Pract*; 2020; **9**: 30–5

251. Jin L, Yao R, Heng L, et al. Ultrasound-guided continuous thoracic paravertebral block alleviates postoperative delirium in elderly patients undergoing esophagectomy: a randomized controlled trial. *Medicine (Baltimore)* 2020; **99**: e19896

252. Lu G-W, Chou Y-E, Jin W-L, Su X-B. Usefulness of postoperative serum translocator protein as a predictive marker for delirium after breast cancer surgery in elderly women. *J Int Med Res;* 2020; **48**

253. Melegari G, Albertini G, Romani A, et al. Why should you stay one night? Prospective observational study of enhanced recovery in elderly patients. *AGING Clin Exp Res;* 2020;

254. Fong TG, Vasunilashorn SM, Ngo L, et al. Association of plasma neurofilament light with postoperative delirium. *Ann Neurol;* 2020; **88**: 984–94

255. Oh ES, Leoutsakos J-M, Rosenberg PB, et al. Effects of ramelteon on the prevention of postoperative delirium in older patients undergoing orthopedic surgery: the RECOVER randomized controlled trial. *Am J Geriatr Psychiatry*; 2021; **29**: 90–100

256. Deng Y, Wang R, Li S, et al. Methylene blue reduces incidence of early postoperative cognitive disorders in elderly patients undergoing major non-cardiac surgery: an open–label randomized controlled clinical trial. *J Clin Anesth*; 2021; **68**

257. Ballweg T, White M, Parker M, et al. Association between plasma tau and postoperative delirium incidence and severity: a prospective observational study. *Br J Anaesth*; 2021; **126**: 458–66

258. Spies CD, Knaak C, Mertens M, et al. Physostigmine for prevention of postoperative delirium and long-term cognitive dysfunction in liver surgery: a double-blinded randomised controlled trial. *Eur J Anaesthesiol*; 2021

259. Humeidan ML, Reyes J-PC, Mavarez-Martinez A, et al. Effect of cognitive prehabilitation on the incidence of postoperative delirium among older adults undergoing major noncardiac surgery: the Neurobics Randomized Clinical Trial. *JAMA Surg*; 2021; **156**: 148–56

260. Wu J, Gao S, Zhang S, et al. Perioperative risk factors for recovery room delirium after elective non-cardiovascular surgery under general anaesthesia. *Perioper Med*; 2021; **10**

## References

[1] Aldecoa C, Bettelli G, Bilotta F (2017). European Society of Anesthesiology evidence-based and consensus-based guideline on postoperative delirium. Eur J Anesthesia.

[2] Inouye SK, Westendorp RG, Saczynski JS (2014). Delirium in elderly people. Lancet.

[3] Hshieh TT, Inouye SK, Oh ES (2018). Delirium in the Elderly. Psychiatr Clin North Am.

[4] Borozdina A, Qeva E, Cinicola M, Bilotta F (2018). Perioperative cognitive evaluation. Curr Opin Anaesthesia.

[5] Bilotta F, Lauretta MP, Borozdina A, Mizikov VM, Rosa G (2013). Postoperative delirium: risk factors, diagnosis and perioperative care. Minerva Anestesiol.

[6] Collaboration AGREE (2003). Development and validation of an international appraisal instrument for assessing the quality of clinical practice guidelines: the AGREE project. Qual Saf Healthcare.

[7] Brouwers MC, Kho ME, Browman GP (2010). Development of the AGREE II, Part 1: Performance, usefulness and areas for improvement. CMAJ.

[8] Brouwers MC, Kho ME, Browman GP (2010). Development of the AGREE II, Part 2: Assessment of validity of items and tools to support application. CMAJ.

[9] Liberati A, Altman DG, Tetzlaff J (2009). The PRISMA statement for reporting systematic reviews and meta-analyses of studies that evaluate healthcare interventions: explanation and elaboration. Br Med J.

[10] Gao R, Yang ZZ, Li M, Shi ZC, Fu Q (2008). Probable risk factors for postoperative delirium in patients undergoing spinal surgery. Eur Spine J.

[11] Large MC, Reichard C, Williams JTB (2013). Incidence, risk factors, and complications of postoperative delirium in elderly patients undergoing radical cystectomy. Urology.

[12] Pinho C, Cruz S, Santos A, Abelha FJ (2016). Postoperative delirium: age and low functional reserve as independent risk factors. J Clin Anesth.

[13] Flink BJ, Rivelli SK, Cox EA (2012). Obstructive sleep apnea and incidence of postoperative delirium after elective knee replacement in the nondemented elderly. Anesthesiology.

[14] Kudoh A, Katagai H, Takase H, Takazawa T (2004). Effect of preoperative discontinuation of antipsychotics in schizophrenic patients on outcome during and after anaesthesia. Eur. J. Anaesthesiol.

[15] Chuan A, Zhao L, Tillekeratne N (2020). The effect of a multidisciplinary care bundle on the incidence of delirium after hip fracture surgery: a quality improvement study. Anaesthesia.

[16] Tai S, Xu L, Zhang L, Fan S, Liang C (2015). Preoperative risk factors of postoperative delirium after transurethral prostatectomy for benign prostatic hyperplasia. Int J Clin Exp Med.

[17] Tognoni P, Simonato A, Robutti N (2011). Re: Preoperative risk factors for postoperative delirium (POD) after urological surgery in the elderly. J Urol.

[18] Mueller A, Spies CD, Eckardt R (2020). Anticholinergic burden of long-term medication is an independent risk factor for the development of postoperative delirium: a clinical trial. J Clin Anesth.

[19] Borchers F, Spies CD, Feinkohl I (2021). Methodology of measuring postoperative cognitive dysfunction: a systematic review. Br J Anaesth.

[20] Laserna A, Rubinger DA, Barahona-Correa JE, et al (2021) Levels of evidence supporting the North American and European perioperative care guidelines for anesthesiologists between 2010 and 2020: A Systematic Review. Anesthesiology 135(1):31-56 10.1097/ALN.0000000000003808 34046679

[21] Tsaousi G, Trombi M, Bilotta F (2021). Comment on: Perioperative dexmedetomidine administration to prevent delirium in adults after non-cardiac surgery: A systematic review and meta-analysis. J Clin Anesth.

[22] Viderman D, Brotfain E, Bilotta F (2020). Risk Factors and Mechanisms of Postoperative Delirium After Intracranial Neurosurgical Procedures. Asian J Anesthesiol..

[23] Bilotta F, Pugliese F (2020). The evolving clinical use of dexmedetomidine. Lancet..

